# Progress in Bacteriology

**Published:** 1893-02-25

**Authors:** 


### CONTEMPORARY THEORY AND RESEARCH.

### PROGRESS IN BACTERIOLOGY.

Professor Lœw points out that Professor Enmerich and Professor Isuboi have investigated the blood of rabbits that were artificially immunised against swine plague (roth lauf). The serum of this blood was (after separation of the globulin) concentrated at 42 deg. Centigrade in vacuo, whereby an albuminous body of prominent curing properties was precipitated. The filtered liquid, however, gave upon precipitation with alcohol also a substance of the same curing qualities. This substance was washed with ether and alcohol, and dried at a low temperature. This dry powder possessed all the curing properties of the blood itself against swine plague. Thus we have for the first time the curing substance (Heilsubstanz) in a dry state, although mixed yet with inactive albuminous substance. This is a fact of immense importance—in bacteriology the most important discovery relating to medicine.

A different branch of this wide subject is treated of in a paper read by Herr Suchland before the German Botanical Society on recent investigations on bacteria found in different kinds of tobacco. Herr Suchland has examined fermented tobaccos from all parts of the world, and found they contain plenty of micro-organisms, although but few varieties, mostly only two or three different species in any particular brand, and but rarely micro-coccus forms. He finds that pure cultures of bacteria obtained from one kind of tobacco and inoculated on to another kind, generated in the latter a taste and aroma recalling the taste and aroma of the tobacco from which the bacteria had been obtained. It is hinted that in the future it may be possible to raise the quality of German tobaccos, not so much by careful culture and judicious selection of varieties, which has so far proved unsuccessful, as by inoculating with pure cultures of bacteria found in some of the fine foreign tobaccos, whereby similar fermentative changes may be induced in the German raw material and the quality correspondingly improved.

